# Challenge to the Intestinal Mucosa During Sepsis

**DOI:** 10.3389/fimmu.2019.00891

**Published:** 2019-04-30

**Authors:** Felix Haussner, Shinjini Chakraborty, Rebecca Halbgebauer, Markus Huber-Lang

**Affiliations:** Institute of Clinical and Experimental Trauma-Immunology, University Hospital of Ulm, Ulm, Germany

**Keywords:** sepsis, innate immunity, gut-barrier dysfunction, perfusion disturbances, enzymatic response, microbiome

## Abstract

Sepsis is a complex of life-threating organ dysfunction in critically ill patients, with a primary infectious cause or through secondary infection of damaged tissues. The systemic consequences of sepsis have been intensively examined and evidences of local alterations and repercussions in the intestinal mucosal compartment is gradually defining gut-associated changes during sepsis. In the present review, we focus on sepsis-induced dysfunction of the intestinal barrier, consisting of an increased permeability of the epithelial lining, which may facilitate bacterial translocation. We discuss disturbances in intestinal vascular tonus and perfusion and coagulopathies with respect to their proposed underlying molecular mechanisms. The consequences of enzymatic responses by pancreatic proteases, intestinal alkaline phosphatases, and several matrix metalloproteases are also described. We conclude our insight with a discussion on novel therapeutic interventions derived from crucial aspects of the gut mucosal dynamics during sepsis.

## Introduction

The mucosa is a highly organized and compartmentalized structure, which lines our body cavities for example, the respiratory, urogenital and intestinal tracts. It provides an interface between the external environment and the host tissues (1), possessing various functions including absorption of water, nutrients and gases, secretion of molecules, clearance of waste, improvement of bio-mechanical features and maintenance of immunity. Therefore, it is not surprising that the combined surface area of the digestive and respiratory tracts by far exceeds the surface dimension of our largest organ, the skin (2). These functions also necessitate a unique immune system which is tightly regulated and this is termed as the mucosal immune system (MIS) (2). The MIS in the gut is capable of distinguishing between regular nutrient flux, self-antigens, a diverse milieu of commensal bacteria and invading pathogenic microbes (3–5). Lymphoid compartments, commonly known as the mucosa-associated lymphoid tissue (MALT), are integrated into the mucosa and perform immune-associated activities. Organized MALT has been found not only in the gut (GALT), but also in a number of other sites, like in the nasopharynx, salivary-gland and duct, larynx, bronchus and urogenital tissues (6). In the intestine, the structural organization of the coexisting symbiotic bacteria is unique, where the large intestine alone houses 10^11^–10^12^ bacteria/gram feces, the highest concentration in the entire intestinal tract (2). However, an imbalance of the co-inhabitation of the intestinal microbiome with the host can potentially threaten well-being (1, 5, 7, 8).

The homoeostatic status quo is essentially supported by the maintenance of the gut barrier integrity. In principle, any infection or severe extra intestinal trauma can cause significant alterations of the gut barrier homeostasis, which may result in a profound generation and secretion of intestinal proteolytic enzymes, alterations in mucus layer formation and composition (9, 10), increased epithelial cell permeability and damaged intestinal cells with subsequent inflammatory signaling (5, 9, 10). These distinct pathophysiological changes are frequently found in septic patients. Previously, sepsis was defined as a systemic inflammatory response (SIRS) with an underlying primary infectious cause (11). Declared as a “silent killer” in critical care units and with high global mortality rates, sepsis has been recently redefined as a “life-threatening organ dysfunction caused by a dysregulated host response to infection” (12, 13). In the clinical setting, diffused and hidden symptoms frequently make the diagnosis of sepsis difficult. To help define septic conditions, clinicians and clinical scientists can utilize the sequential (sepsis-related) organ-failure assessment (SOFA) Score, which allows more precise detection of sepsis-associated organ dysfunction compared to the SIRS-criteria (12–15). The alarming pace of sepsis with possible development of multiple organ dysfunction syndrome (MODS) frequently includes disseminated intravascular coagulopathy (DIC), making sepsis patients a colossal challenge for both clinicians and researchers.

Years of research have focused on the various intricacies, from the underlying pathology to clinical targets that could help treat sepsis patients. In the scope of our review, we consider the effects of sepsis on the intestinal mucosa regarding the main immunological mechanisms that yield a dysregulated intestinal mucosal system and the scope of associated promising therapeutic strategies.

### Structure-Function Relationship of the Intestine for the Maintenance of Immune Defense

The surface of the *small intestine* is formed by a monolayer of highly prismatic epithelia, which are modified into structures like plications, villi (0.2–1 mm), crypts, and microvilli. Crypts contain stem cells, which generate intestinal epithelial cells (IECs). Paneth cells within the crypts secrete antimicrobial peptides (AMPs), for example, α-defensin and lysozyme, to confer intestinal protection from pathogenic insults (16, 17). The IECs in villi reabsorb nutrients and are interconnected by tight junctions (TJs) (e.g., occludins, claudins) that form apical paracellular seals thus preventing the flux of hydrophilic molecules (18). Further along the IECs lie adherens junctions (e.g., cadherins) and gap junctions (e.g., connexins), all of which determine the cellular polarity and regulate cell-cell communication and exchange of substances. The epithelium can also secrete pro-inflammatory cytokines and reactive oxygen species (ROS) in response to pathogens and metabolic stress (19). Goblet cells in the villi produce mucus, a key component of the gut barrier. A single unattached mucus layer is present superficially on the surface of the small-bowel epithelia (20, 21). Mucus contains soluble glycoproteins termed mucins, which are normally negatively charged, consisting of a core protein to which multiple polysaccharide moieties are attached, capable of binding water molecules (22). In addition to the predominant mucin-2 (MUC2), other bioactive molecules, for example, membrane-bound mucins, like MUC1, MUC3, and MUC17 and peptides, like Fc-γ binding protein and intestinal trefoil factor peptides, are secreted by goblet cells (22, 23). These play a major role in maintaining mucosal homoeostasis, mainly by limiting contact between commensals/pathogens and IECs (23). The *large intestinal mucosa* comprises crypts without any villi, with significantly greater numbers of goblet cells in comparison to the small bowel. The colon functions mainly as a reabsorbing organ for water and electrolytes and additionally produces mucus. One important distinction is the double layer of mucus on the colonic epithelial cell surface, where the inner layer is immediately above the epithelium, is mostly immobile and is thinner than the outer mucus layer, which is not attached to the colon wall (24). Both layers consist of gel-forming MUC2, but the glycoproteins of the inner layer form a large and dense net, whereas the outer layer consists predominantly of MUC2 cleavage products (25).

**Figure 1:**
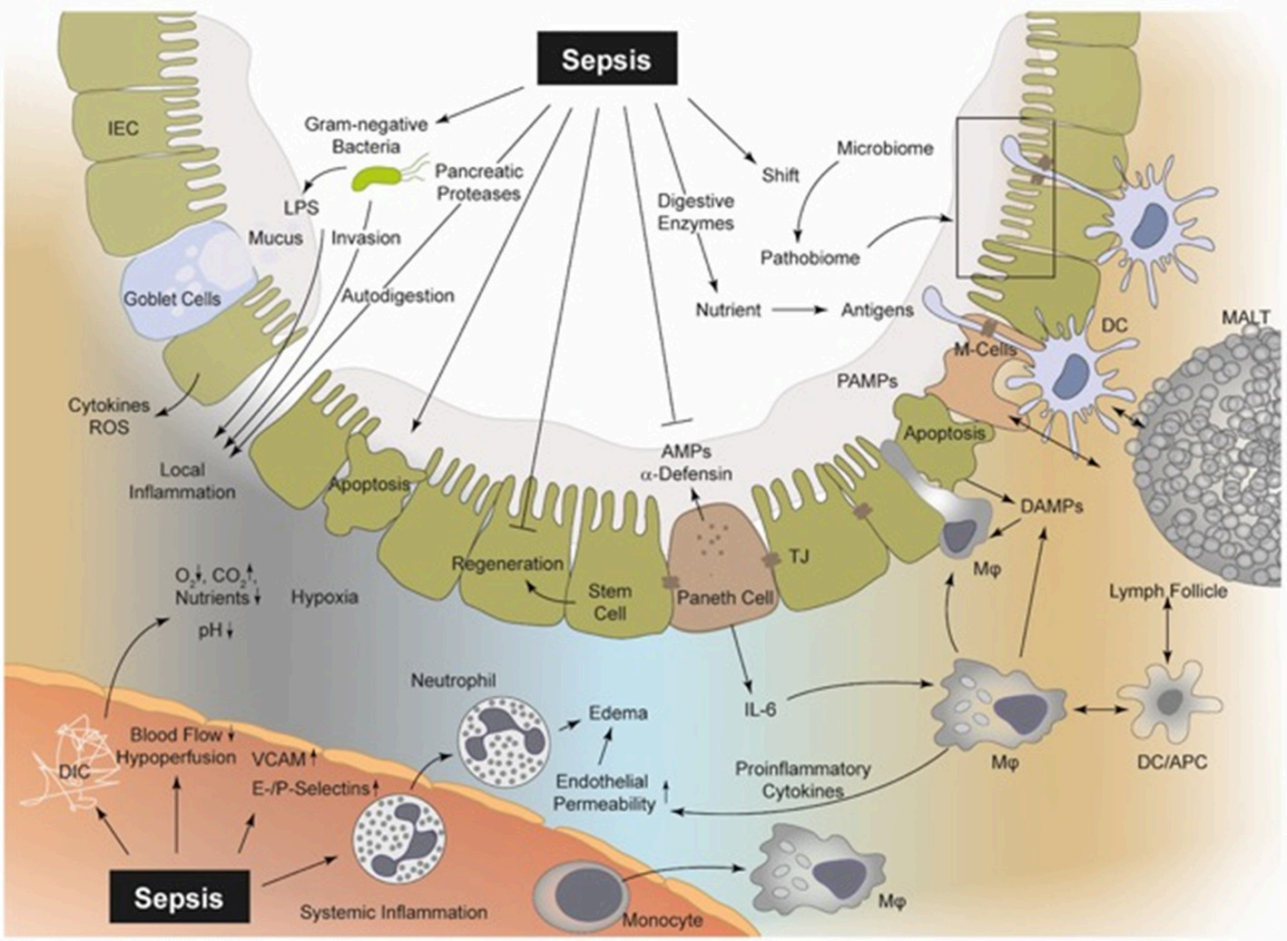
Sepsis is involved in several pathophysiological processes regarding the intestinal epithelial integrity, perfusion, coagulation, enzymatic response, and MIS. In sepsis, bacteria and their products (PAMPs), including LPS, PG, and bacterial DNA, can be recognized by PRRs (e.g., TLR2 and TLR4) upon the surface of macrophages, neutrophils, DCs, and even IECs (19, 32). Thereby, intestinal macrophages and DCs as part of the MIS can detect luminal PAMPs via transepithelial dendrites (TEDs) (26, 27, 33). Consequently, PAMPs induce a “cytokine storm” of pro-inflammatory mediators, which drive the local intestinal and systemic inflammation (32). The released mediators can lead to an upregulation of endothelial adhesion molecules (e.g., ICAM, VCAM, E-, and P-selectin), resulting in increased recruitment of neutrophils and monocytes and in turn to increased levels of pro-inflammatory cytokines and ROS (34, 35). These cellular responses aggravate vasodilatation and induce a high level of capillary leakage with the development of interstitial edema. Local DIC is frequently observed during sepsis with a decreased supply of oxygen and nutrients, but increased carbon dioxide concentration (36, 37). Hypoxia in turn leads to increased apoptosis and necrosis of IECs and the regeneration of these IECs is suppressed during sepsis (38–40). Furthermore, the IEC integrity is disrupted and bacterial translocation may be facilitated. Pancreatic proteases are capable of autodigestion and potentiation of MOF and self-digestion leads to an increased release of further DAMPs (10, 19, 41, 42). MIS, mucosal immune system; PAMPs, pathogen-associated molecular patterns; DAMPs, danger-associated molecular patterns; LPS, lipopolysaccharide; PG, proteoglycan; PRR, pattern-recognition-receptors; TLR, toll-like receptor; DCs, dendritic cells; IECs, intestinal epithelial cells; TEDs, transepithelial dendrites; DIC, disseminated-intravascular-coagulation; MOF, multi-organ failure; ICAM, intercellular adhesion molecule 1; VCAM, vascular cell adhesion protein 1; ROS, reactive oxygen species; M-cells, microfold cells; AMPs, antimicrobial peptides; APC, antigen presenting cells; TJ, tight junctions; MALT, mucosa-associated-molecular pattern.

Regarding cellular immunity in the intestine, there is a well-regulated interplay between antigen-presenting dendritic cells (DCs), intestinal macrophages and adaptive immune cells. After recognition of antigens and/or pathogen-associated-molecular-patterns (PAMPs) via pattern recognition receptors (PRR), including Toll-like-receptors (TLRs) and NOD-like-receptors, intestinal DCs regulate the immune response by enhancing or suppressing T-cell activity. To achieve this, dendrites of DCs penetrate intercellular spaces through the intestinal TJs while maintaining barrier integrity (26). DCs, via these dendrites sense and bind luminal PAMPs and bacteria and present processed antigens to immune cells located in lymphoid follicles found in the connective tissue and the lamina propria. Intestinal macrophages (type CX3CR1^hi^) can also sense PAMPs by forming transepithelial dendrites (TEDs). Of note, this specific type of macrophage has only been observed in the murine ileum and the importance of the TEDs remains uncertain (27). Another means to reabsorb antigens is accomplished by villous microfold cells which offer antigens a channel to lymphoid tissue, where antigen presenting cells resorb the molecules and present them to CD4^+^T-cells via Major-Histocompatibility-Complex II (28). Moreover, DCs selectively induce a pro- or anti-inflammatory immune response by interacting with T- and B cells. IgA^+^-B cells colonize in the lamina propria and secrete IgA into the lumen via transcytosis (29–31) (Figure 1). This complex intestinal organization is subject to activation and dysregulation during sepsis.

### Gut Barrier Dysfunction and Systemic Consequences During Sepsis

A major pathophysiological mechanism of sepsis harnesses recruitment of inflammatory cells and generation of an overwhelming pro-inflammatory response. PAMPs, for example, lipopolysaccharide (LPS), peptidoglycan and bacterial DNA among others and damage-associated molecular patterns (DAMPs), including mitochondrial DNA, High-Mobility-Group-Protein-B1 and serum amyloid A, result in the upregulation of adhesion molecules on the intestinal endothelium followed by the recruitment of neutrophils and macrophages (43). Upon migration to the intestinal tissue, these cells of the first line of defense produce pro-inflammatory cytokines, clinically manifested as classical signs of local and systemic inflammation (32, 44). Cell-wall components from gram-negative and gram-positive bacteria activate PRRs like TLR4 and TLR2, respectively, resulting in a “cytokine storm” of pro-inflammatory mediators generated mainly via the mitogen-activated protein kinase and NF-κB pathways (32). Of note, pro-inflammatory responses are interspersed with anti-inflammatory responses, also termed the compensatory anti-inflammatory response syndrome (45–47), where patients with sepsis undergo a reprogramming of their defense strategies and frequently fail to eliminate primary infection, thus being unable to prevent secondary infection development (33). However, an initial hyper-inflammatory response might dominate to beneficially isolate local infectious foci and limit systemic spillover (33). Gut barrier dysfunction can be considered both a result and a cause of sepsis development, characterized by enhanced mucosal layer permeability (5, 9, 10, 23, 48–51), disturbed mucosal perfusion (38, 52–54), development of tissue edema, coagulation-associated local dysregulation (36, 37), bacterial translocation (48, 55, 56) and a shift in the gut microbiome (57, 58). Furthermore, apoptotic and necrotic mechanisms damage the mucosal epithelia, resulting in a vicious cycle of further release of DAMPs, feeding into inflammatory responses combined with the development of ulceration and hemorrhage and exacerbation of mucosal homeostatic imbalance (Figure 1) (50, 59). The causes and consequences of gut barrier dysfunction have been described in literature extensively.

### Disturbances in Vascular Tonus and Perfusion

Hypoperfusion in the splanchnic region is considered one of the main reasons for mucosal gut barrier breakdown during sepsis (38). The splanchnic vasculature system normally receives about 25% of the total cardiac output, which increases up to 35% during digestion (60, 61). Perfusion is mainly controlled by local mediators, including nitric oxide and prostaglandin derivatives, but also by systemic mediators, like vasoactive substance P and by the sympathetic innervation (61). Splanchnic hypoperfusion converts the gut into a cytokine-generating organ, which releases a “toxic fluid,” containing pro-inflammatory agents and induces MODS via the circulation (48). Hypovolemia and cardiac depression during sepsis are associated with a robust inflammatory response of cytokines and other inflammatory mediators (39). Blood cells, endothelium and vascular smooth musculature are potential targets of these pro-inflammatory cytokines leading to vasodilatation, high capillary leakage, increased venous capacity and decreased venous return, all of which result in a decrease in cardiac output and tissue perfusion (39, 40). In turn, the renin-angiotensin-aldosterone-system is stimulated and increasingly generates vasoconstrictive agents, which also adds to local hypoperfusion thus developing both micro- and macro-circulatory disturbances (39, 52, 62–64). As an overall consequence, gut mucosal perfusion is reduced during sepsis, which results in further hypoxia and consequent destruction of the mucosal barrier (38). Studies using laser Doppler measurements have also revealed that CLP-induced sepsis in normotensive rats caused a decrease in the number of perfused capillaries in the small gut mucosa (65). In this context, it is also known that mucosal blood flow is dependent on inflammatory processes (52). As a result of sepsis-associated excessive inflammation, the microvasculature loses its capacity to regulate blood flow and oxygen distribution mainly based on the generation of ROS (39, 40, 66). As a consequence, increasing the blood flow by vasodilatation during hypoperfusion is not possible (53, 54), explaining why maximal O_2_ extraction cannot be accomplished in sepsis (Figure 1). A further process that results in an impairment of the local vasodilatory response is the pathological opening of arteriovenous shunts that alters the blood flow between hypoperfused and perfused areas (39, 67).

There exist further sepsis-induced alterations in perfusion, including increased intercapillary distances due to edema (39) and greater diffusion distances (39, 63). However, the pathophysiological details are beyond the scope of this review.

### Increased Intestinal Permeability

**Figure 2:**
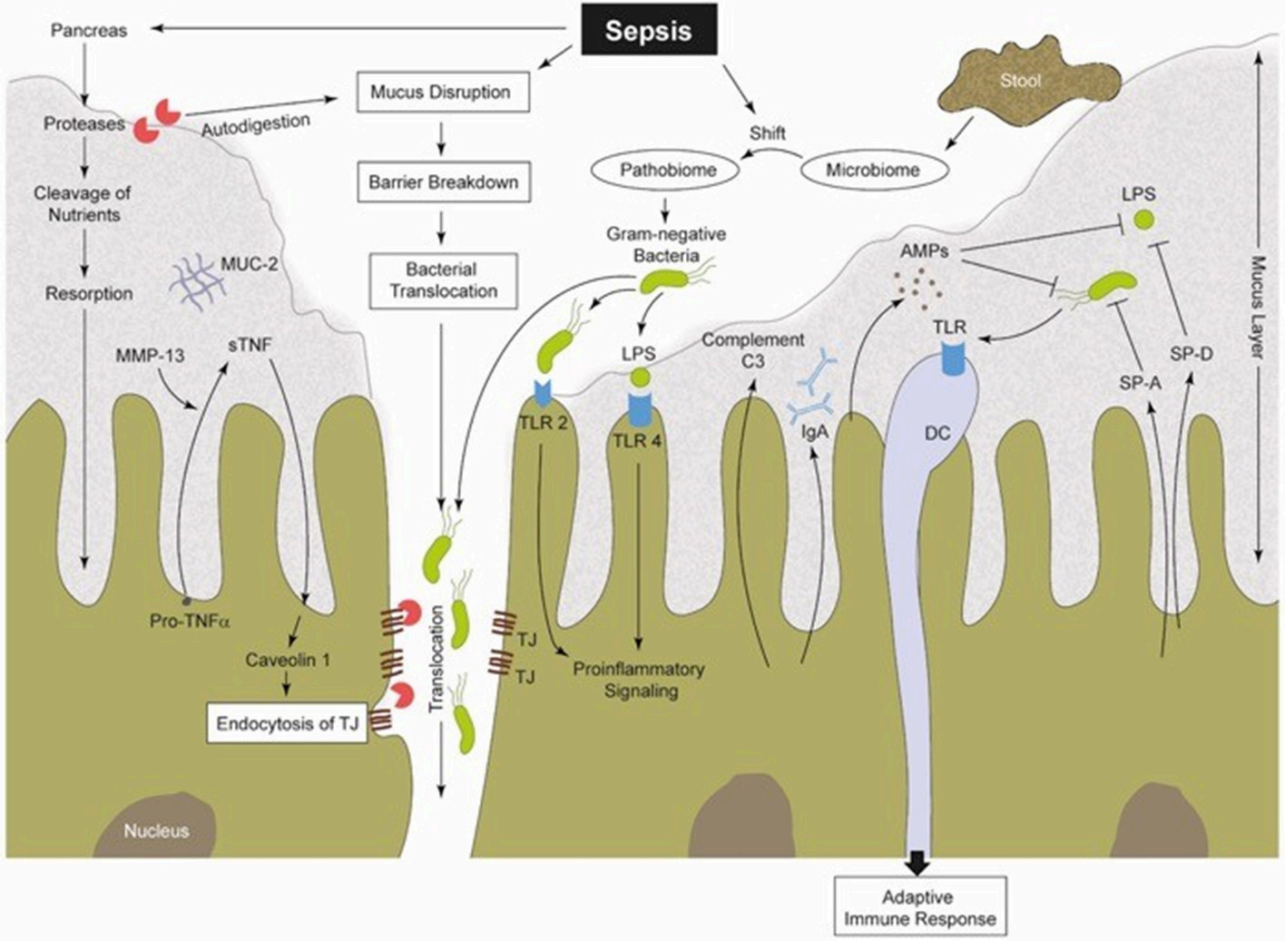
Sepsis-induced alterations in perfusion, vascular tonus and coagulation lead to a hypoxic microenvironment of the intestinal tissue (38). Therefore, the protective gel-forming MUC2 mucus layer becomes disrupted (22). Both, bacteria (products) and (pancreatic) proteases gain access into intestinal epithelia, inducing damage followed by increased pro-inflammatory signaling (41, 48, 70, 70, 71). Furthermore, MMPs (like MMP13) are able to cleave membrane-bound pro-TNF into sTNF, which in turn is able to stimulate caveolin-1-dependent endocytosis of TJs (72, 73). Gut barrier breakdown and dysfunction is one consequence (72). Intestinal commensal microbes regulate the maturation of the MIS and support local mucosal immunity (8, 74). During sepsis, the well-regulated interplay between the commensal microbiome, IECs and mucosal immune cells becomes imbalanced. There is a sepsis-induced shift from a physiological microbiome to a “pathobiome,” which is able to dysregulate the immune system by activating PRRs (32, 58). SP-A and SP-D can be synthetized by IECs (75, 76). These SPs are capable of increasing the permeability of bacterial membranes and in turn reduce the bacterial burden (77). IECs produce AMPs (e.g., α-defensin and lysozyme), to confer intestinal protection from pathogenic insults. Thereby, AMPs can act two ways, on the one hand directly by antimicrobial killing and on the other hand by innate immune modulation (78). Complement factors are mainly produced in the liver (79, 80), but also IECs were identified to synthesize and secrete C3 into the intestinal lumen (81), and thus may also play a role in intestinal immunity (82, 83). IECs, intestinal epithelia cells; MUC2, mucin-2; MMPs, matrix metalloproteinases; TNF, tumor necrosis factor; sTNF, soluble TNF; TJs, tight junctions; MIS, mucosal immune system; PRR, pattern recognition receptors; TLR, toll-like receptor; SP, surfactant protein; AMPs, antimicrobial peptides; C3, complement factor 3; LPS, lipopolysaccharide; DC, dendritic cell; IgA, immunoglobulin A.

It is well-established that sepsis results in a dysfunction of the intestinal barrier with increased permeability (5, 9, 10, 23, 48, 49, 51, 68, 69). Locally transmigrated bacteria and endotoxin exposure lead to a local activation of the MIS and in turn to the production of various pro- and anti-inflammatory cytokines by IECs and intestinal immune cells. This cellular response may also contribute to the systemic response (Figure 2). Furthermore, activation of intestinal immune cells results in a further increase in gut permeability by altering TJs (68, 69, 84, 85). Induction of experimental sepsis leads to a redistribution of the TJ proteins occludin and claudin-1, 3, 4, 5, and 8 (68, 85). In agreement with this, murine endotoxemia resulted in a disrupted ultrastructure of occludin and zonulin-1 (ZO-1) in the intestinal epithelium (86). Of note, these changes could be corrected by vagal nerve stimulation (86) or even by treatment with plant products, including berberine (87). Berberines are known to decrease downstream myosin-light chain kinase (MLCK) and NF-κB activity, representing mechanistic intermediates that may modulate TJ organization. Elaborating on this mechanism, it was found that MLCK phosphorylates myosin light chain which causes cytoskeletal contraction and junction disruption (88). Furthermore, β-catenin, another TJ organization protein, was found to be irregularly distributed in LPS-treated rats while platelet activating factor appeared to attenuate this disorganization (89). More recently, increased plasma ZO-1 levels have been found during experimental sepsis and an elevated plasma zonulin concentration, a regulator of TJs, in patients with sepsis (90). Cyclooxygenase-2 (COX-2) and particularly its product prostaglandin D2 appear to play an important role in the maintenance of epithelial TJs and barrier function, because the absence of COX-2 led to an increased permeability of the murine ileum and to a reduced expression of TJ proteins (91). Consequently, reinforced bacterial translocation and a higher mortality rate were observed in septic COX-2-knockout mice after cecal ligation and puncture (CLP) (91). With enhanced permeability, what becomes entirely imminent is the translocation of bacteria, a highly probable threat to the intestinal mucosal system.

### Bacterial Translocation via the Mucosa

Gut permeability theoretically prompts the possibility of local bacterial translocation, supported by several pieces of evidence. For example, TLRs play a role in directing the response via MLCK activation, as shown in morphine-treated animals, which in turn facilitate bacterial translocation and even cause infection or sepsis of gut origin (92). Other factors like insulin growth factor-1 promote bacterial translocation, which induces intestinal cell apoptosis (93), or increased pneumoperitoneal pressure (during laparoscopic surgery) (94). Sepsis development has also been reported as a secondary effect of pneumonia due to mucosal and microvascular injury in the gut (95). In turn, bacterial translocation may occur as a common process, secondary to a primary infection, severe trauma, or major surgery, giving rise to sepsis and consequently supporting the “gut origin hypothesis of sepsis” (96).

There are further assumptions about the driving force of sepsis and MODS. Translocating bacteria and endotoxin from the gut lumen may not directly enter into the systemic circulation, but rather induce an immune response in the local GALT or draining lymph nodes, which results in significant systemic effects, for example, via “toxic lymph” (Figure 2) (48, 70). The mesenteric lymph contains several different proteins and lipid factors, including a modified albumin species (97), which could cause cellular damage and the activation of TLR4, resulting in priming of neutrophils and inducing remote lung injury (98). In agreement with this, a correlation between gut barrier dysfunction and secondary lung injury has been found (48, 49). Because novel techniques on the nanoscale in bacterial (product) detection have been developed over the last decade, further clinical studies may help to re-evaluate and elucidate the “bacterial translocation” paradigm and its mechanisms.

### Coagulation and Its Factors Modulating Mucosal Dysfunction

The mechanisms of sepsis-induced consumptive coagulopathy are manifold. The procoagulant tissue factor (TF), which is produced by the liver, monocytes, neutrophils and endothelial cells, is significantly increased after exposure to endotoxin or PAMPs (36, 99, 100). Synchronous inhibition of fibrinolysis occurs by an enhanced production of plasminogen activator inhibitor (PAI-1) and the downregulation of the protein-C pathway, which are important in the initiation and progression of coagulopathy with clinical manifestation of both thrombosis and DIC (36, 37). While intestinal microcirculatory disturbances are common during sepsis-induced DIC, various clotting factors may directly or indirectly affect intestinal physiology and immune-cell recruitment. For example, septic rats displayed a decrease in functional capillary density, indicating a reduction in microvascular perfusion, which could be corrected when these animals were treated with factor XIII (FXIII) (101). In addition, factor-XI deficiency could confer a survival advantage on mice with peritoneal sepsis (102). In agreement with this, microcirculatory disturbances were found in the intestinal epithelium of CLP rats associated with high intestinal TF levels, all of which could be improved by sodium tanshinone IIA sulfonate, a substance recently proposed to exhibit protective effects against coagulatory disturbances (103). Similar effects were shown for the thrombin inhibitor Argatroban (104). Dual pharmacological inhibition of factor II and factor Xa by SATI resulted in preserved activation of coagulation with no bleeding complications and protection of organ function during experimental sepsis in baboons, representing a promising tool against sepsis-induced DIC (105). Furthermore, treatment with recombinant human antithrombin has been shown to ameliorate leukocyte adhesion in mesenteric venules and to reduce intestinal injury in endotoxemic rats (106) and concomitantly improved the 28-day mortality rate in septic patients (107). Nevertheless, application of PAI-I (108) or recombinant human thrombomodulin (109) failed to reveal beneficial effects in septic patients. Linking mucosal immunity to coagulation, mucosal M2 macrophages have been recently shown to contain intracellular FXIII stores. The cell number of this subtype is decreased in inflamed mucosa in the setting of ulcerative colitis (110). Previously, macrophage procoagulant activity was found to be increased in rats with depleted intestinal microflora and orally fed with streptomycin-resistant *E. coli*, implying that the gut is a focal point from which systemic inflammation arises (111). Deficiency of carboxypeptidase B2, an enzyme able to cleave both fibrinogen and the central complement components C3 and C5, was shown to confer survival advantage to mice, which was mainly mediated by C3a-induced peritoneal macrophage recruitment (112). Although there is evidence of an intensive crosstalk between coagulation and the innate immune response in driving inflammation during sepsis, the exact underlying mechanisms still need to be defined.

### Apoptosis as a Central Driver of Intestinal Damage

Apoptotic events play a critical role in the development of sepsis. Interestingly, in murine sepsis models and in autopsy studies of septic humans, there were barely any significant histological changes except for increased gut epithelial/lymphocyte apoptosis in comparison with non-septic deceased patients (113). Nevertheless, experimental prevention of apoptosis in sepsis models increased the survival rate (33, 113) and therefore, the hypothesis of immune cell apoptosis as a relevant pathological mechanism in sepsis could also be of special interest for mucosal immunity (114). Sepsis induced by *Pseudomonas aeruginosa* pneumonia was, for example, caused by apoptotic intestinal epithelia associated with reduced epithelial proliferation (45). Mechanistic investigation of intestinal cell apoptosis during sepsis identified gene overexpression of interleukin (IL)-1β-converting enzyme, which may play an important role during experimental sepsis (115). In addition, when anti-apoptotic proto-oncogene Bcl-2 was gut-specifically overexpressed, a decrease in sepsis-induced intestinal epithelial apoptosis was found in murine models (45, 116, 117). MicroRNA 195, a regulator of Bcl-2 gene expression, which assists in maintaining the pro/anti-apoptotic balance, has been shown to be upregulated in murine sepsis and its inhibition could prevent apoptosis and even the development of MODS (118). Therefore, new approaches to improve gut barrier function during sepsis could be represented by application of silencing microRNAs regulating intestinal apoptosis (117, 118).

Apart from generic apoptosis related transducers, other molecules have also been implicated to play a role in sepsis-associated apaptotic mechanisms. Cytokine IL-15 was identified to be capable of preventing apoptosis and of immune suppression as well. In sepsis, IL-15 attenuated the apoptosis rate of intestinal epithelia and increased Bcl-2 and IFN-γ expression in IECs as well as the natural killer cell population, which produced further IFN-γ (119, 120). Of note, the lung surfactant proteins SP-A and SP-D have additionally been found to be generated by epithelial cells of the small and large intestines and in gastric cells (75, 76), and the absence of SP-A and -D resulted in increased LPS-induced apoptosis of primary IECs (77). Nonetheless, to what extent surfactant molecules may therapeutically protect the gut barrier remains to be investigated.

### Intestinal Microbiome as an Actor and Target

During the last decade, the commensal microbiome has been defined to play a key role in intestinal immunity because microbes regulate the maturation of the MIS (8, 74), support local mucosal immunity (7, 8) and regulate cellular growth and maintenance of epithelial barrier function (1, 5). It is likely that the human immune system not only controls bacteria, but that the microbiome also regulates the immune cell function, particularly on mucosal surfaces (8, 121). It putatively modulates neonatal immunity and determines susceptibility to infection depending on the mode of childbirth (122–125). Alterations of the lung microbiota due to colonization by gut microbes has also been shown in animal studies, which to some extent may explain the frequent simultaneous appearance of acute respiratory distress syndrome with sepsis (126). If the symbiosis between commensal bacteria and the human host becomes imbalanced, the innate and adaptive immune systems are disturbed (Figure 2) (121, 127). A decline or even a loss of protective anaerobes in fecal specimens has been observed in patients with severe sepsis (57, 58) and hypothetically this “pathobiome” is able to manipulate and dysregulate the immune system in critically septic and ill patients (58). Moreover, commensal bacteria are involved in the regulation of CD4^+^ T-cell immunity though the exact mechanisms remain unknown (128). Indicating the harmful effect of opioid analgesics in treating critical care patients, murine polymicrobial sepsis with opioid treatment selectively influenced gram-positive gut microbiome translocation and dissemination, inducing its pro-inflammatory effects through IL-6 and IL-17A cytokines (129). Furthermore, the function and aging of neutrophils as first cellular line of defense were also shown to be regulated by the microbiome during sepsis (130, 131). Overall, it is tempting to speculate that therapeutic interventions on the altered microbiome might improve barrier, immune and organ function as well as sepsis outcome.

### Intestinal Enzymatic Response Induces Self-Destruction

The underlying mechanisms of the interplay between pancreatic enzymes, sepsis, and septic shock remain unclear, although Schmid-Schönbein and colleagues had already hypothesized in 2005 that pancreatic enzymes are capable of self-digestion and potentiation of multi-organ failure (MOF) (41). In the case of sepsis-induced hypoperfusion/ischemia of the intestine, autodigestion processes can affect the mucosal barrier (10, 42). Such self-digestion may lead to an increased release of DAMPs and enhance the systemic response due to the release of pro-inflammatory mediators by stressed IECs (Figure 2) (19, 42, 71). The inhibition of pancreatic enzymes with subsequent prevention of gut-specific autodigestion indeed improved the outcome of septic mice (132). In this regard, inhibiting pancreatic proteases with tranexamic acid reduced inflammation and could also be exploited as a future sepsis treatment beyond its application in treating traumatic coagulopathy (132). Intestinal alkaline phosphatase (IAP) is another enzyme that protects the intestinal brush borders, particularly against intestinal bacterial invasion (133). Some of the major functions of IAP are duodenal surface pH regulation (via ${\text{HCO}}_{3}^{-}$ secretion), mitigation of intestinal inflammation by PAMPs and gut microbiome control (134). IAP-mediated inactivation of bacterial products, including LPS, decreases their binding to TLR4 and reduces the resultant inflammatory responses. Interestingly, in the absence of bacteria, a lack of IAP expression results in the loss of mucosal protection (135). Mice treated with IAP after exposure to a lethal dose of *Escherichia coli* had an improved survival rate of 80%, compared to 20% in the control sepsis group (135, 136). In conclusion, loss of IAP expression or function increased intestinal inflammation, dysbiosis, and bacterial invasion, culminating in systemic inflammation (134).

LPS can furthermore induce matrix metalloprotease 7 (MMP7) expression and degranulation of Paneth cells, leading to increased intestinal permeability (17). MMP7 was observed as an amplifier of inflammation; MMP7-deficient mice displayed an attenuated intestinal inflammatory response (137). MMP7 is able to activate α-defensin, which in turn stimulates IL-6 release by macrophages and ileal epithelia, thereby enhancing local intestinal inflammation and damage (137). Moreover, MMP7 has also been correlated with the loss of intestinal barrier integrity, enhanced bacterial translocation and MOF development (137). Similarly, MMP13 has been described to play a role in inflammatory bowel diseases (IBD) and during sepsis (72). It is able to cleave membrane-bound pro-TNF into soluble bioactive TNF, which can affect TJs through caveolin-1-dependent endocytosis (72). The consequences are the loss of TJs, increased intestinal permeability and the creation of a new pathway for migrating bacteria, which induces further inflammation (72, 73, 138). MMPs are also present in the large intestine and play a similar role in sepsis progression through similar mechanisms. MMP-1, 2, 3, and 9 were detected in the human colon mucosa and were also increased during IBD (139, 140). However, their exact role in sepsis is yet to be investigated.

### Metabolic Response Within the Intestinal Mucosa

While intestinal permeability is enhanced, amino-acid absorption by the intestine is affected as early as 24 h after sepsis onset (141). In this regard, *in vivo* and *in realiter* studies revealed that gut glutamine absorption and metabolism decreased during sepsis because of suppressed glutaminase activity (142). By contrast, glutamine supplementation improved other effects of sepsis: it reduced bacterial translocation, restored permeability and microcirculatory characteristics (143–146), and even increased the number and survival of intestinal epithelia while blocking inflammatory cytokine secretion by CD8αα(+) TCRαβ(+) IEL cells (147) and γδT-IELs (148). As a further risk factor, a high-fat diet was detrimental for sepsis outcome and worsened endotoxemia in mice by disrupting the *Bifidobacterium spp*. colony. Correction of the dysbiosis and its consequences by feeding prebiotic oligofructose resulted in reduced systemic inflammation in experimental (149) and clinical sepsis (150). This preliminary evidence suggests that the intricate repertoire between metabolic intermediates, gut microbiome and inflammatory responses following sepsis requires further investigation and represents a promising therapeutic potential.

### Therapeutic Approaches to Improve Sepsis-Associated Mucosal Immunopathology

In the era of resurrection from the “therapeutic graveyard of sepsis,” novel pharmacological approaches address crucial aspects of gut mucosal dynamisms. For example, the dietary dipeptide gamma-l-glutamyl-l-valine (γ-EV), which leads to decreased pro-inflammatory cytokines in both plasma and the small intestine, is also effective against bacterial infections (151). γ-EV stimulates the interaction of β-arrestin-2 with toll-interleukin-1-receptor signaling proteins, including TRAF6, TAB1, and IκBα, which further suppress the inflammatory response in the small intestine (151, 152). Similar results in murine IBD models have been shown for γ-glutamyl-cysteine, which inhibits TNF signaling in intestinal epithelia (152). In other studies, the small peptide hormone ghrelin was identified to be protective by inducing autophagy in the case of tissue hypoxia. Thereby, ghrelin appears also able to protect IECs in the small intestine in the early stage of sepsis (153). Application of deacetylase sirtuin-1, a signaling intermediate that is decreased in obesity and results in enhanced microvascular inflammation within the small intestine, reduced the mortality rate in the early stage of sepsis (154, 155). Treatment with resveratrol increased the expression of sirtuin-1 in obese septic mice and the inflammatory response thereafter was diminished (154). Sirtuins also play a major role during the late onset of septic “hypo-inflammation”; SIRT-2 inhibition in obese septic mice preserved a decreased microvascular inflammation and protected against thrombotic events (155).

The antimicrobial peptide cathelicidin-BF (C-BF) has been observed as a protective molecule, which can safeguard LPS-induced septic rodents from the development of small intestinal barrier dysfunction (156). C-BF prevented LPS-induced TJ breakdown and reduced IEC apoptosis by attenuated expression and secretion of TNF and suppression of the underlying NF-κB pathway (156). Ulinastatin is another drug able to increase the survival rate and to reduce injury of the small intestine, for example, through diminished IEC apoptosis (142). Post-treatment IL-6 and TNF plasma levels were decreased, suggesting an interesting strategy for sepsis (59, 157).

Stem-cell therapy could also represent a potential treatment approach for sepsis. In murine CLP-sepsis, human adipose-derived mesenchymal stem cells were able to modulate sepsis by downregulation of Th1-cell responses, associated with lower levels of pro-inflammatory cytokines (TNF, IL-1β, IL-6, IL-12, IFNγ) and higher levels of anti-inflammatory IL-10 derived from macrophages (158). Application of (mesenchymal) stem cells may, therefore, protect septic mice by reducing inflammatory cell infiltration and pro-inflammatory responses and enhancing anti-inflammatory signals (158). Nevertheless, to what extent stem cells or their cellular structure or secretome will modulate mucosal immunity during sepsis has to be clarified in future studies.

Defining the undisputed role of the microbiome in shaping sepsis-associated immunopathology is gradually gaining momentum, discussed in detail in several reviews (159–161). A dysregulated gut microbiome is a common causation of sepsis (162), like in late-onset sepsis development of preterm neonates (163). Conversely, burn-injury associated altered gut microbial community and leakiness of the gut have been implicated in sepsis development (164). A common approach undertaken to manage sepsis patients involving therapy with antibiotics can impair the diversity of microbes in the intestine and reduce the protective role of bacteria, which in turn leads to increased inflammation in murine models of Gram-positive as well as Gram-negative pneumosepsis (7, 165, 166). Thus, the loss of microbiome diversity was indeed identified as a predictive factor for the length of hospitalization of patients in the ICU (166, 167). Though a recent study disproved that antibiotics-mediated disrupted microbiota modulates innate immune system in endotoxemic patients (168), the exact role of how the immune system is modulated is left to be delineated. Nevertheless, to correct this ensuing dysbiosis, treatment options have included procedures like fecal microbiota transplantation (FMT) with combined usage of antibiotics in the clinical management of sepsis (169–171). In the 1950s, FMT had been developed to treat *Clostridium difficile* associated pseudomembranous colitis and has since subsequently proven to be an effective treatment modality in the management of *C. difficile* infection (172–174). Recently, the US Centers for Disease Control indicated that “death rates from sepsis following infections (e.g., *C. difficile*) have surged (175). Therefore, a perfect premise to facilitate new treatment approaches, FMT is a potentially effective treatment route, which could counterbalance dysbiosis, support the gut microbial barrier and improve the outcome of sepsis (171, 172, 176). Microbial dysbiosis of the gut leads to changes in the metabolism of bacteria and as a consequence to an impaired interaction between microbes, immune cells and IECs (1, 5, 7, 8). During sepsis, the exact mechanism of action for the use of FMT on the intestine is still unknown. Recolonization of the intestinal microflora has been beneficial as well, where 16 days post-FMT, improved symptoms were observed in two separate patient studies involving stroke (176) and post-surgical sepsis development (171). FMT proves to be a viable future treatment option for sepsis and further human clinical research is needed to evaluate its effectiveness in critically ill patients.

Further therapeutic approaches have diversified to include supplementing antibiotics with probiotics, prebiotics and synbiotics. Probiotics are live beneficial microorganisms, which can improve the health of hosts (170), prebiotics are non-viable and non-digestible dietary ingredients e.g., fructooligosaccharides which stimulate the growth and/or activity of a limited number of bacteria in the large intestine (170, 177) and synbiotics refer to combined usage of prebiotics with probiotics (178). Studies have shown that supplementation of *Bifidobacterium breve* strain Yakult and *Lactobacillus casei* strain Shirota as probiotics and galactooligosaccharides as prebiotics can reduce the incidence of infectious complications, e.g., enteritis, pneumonia and bacteremia in patients with severe SIRS compared to those who did not receive synbiotics (179, 180). The administration of synbiotics could maintain the gut flora and reduce septic complications in patients with severe SIRS by enhancing the levels of beneficial bacteria in the intestine. A further study suggests that the orally consumed synbiotics (*Lactobacillus planatrum* and fructooligosaccharide) in newborn infants improve the primary outcome (complication of sepsis or death) as well as lower respiratory tract infections compared to newborn infants with placebo treatment (181). In contradiction to these studies, other studies have suggested no difference in incidence of late-onset sepsis and mortality rate in preterm infants (182, 183). Similar results postulated that prophylactic administration of *B. clausii* to preterm neonates do not reduce the burden of late-onset sepsis compared to placebo (184). Further, research may be focused on dysbiosis of the gut microbiome and resultant immunosuppression as one consequence of sepsis restored by gut commensals through administration of probiotics, to reduce the incidence of late infections and the sepsis mortality rate (185, 186). Synbiotics also seem to be a potential treatment option for sepsis patients. The complications of enteritis and ventilation-associated pneumonia were significantly lowered in the patients who have been treated with synbiotics, compared to those without synbiotic administration, although the incidence of bacteremia and the mortality rates did not differ between the groups (169). The process of bacterial translocation from the intestinal lumen to systemic circulation as described in section Bacterial Translocation via the Mucosa is another interesting premise to consider as a treatment target. There are some clinical correlations showing bacterial translocation as one cause for subsequent sepsis, or in reverse, induce late onset-sepsis complications. In patients with acute pancreatitis, septic complications as a result of pancreatic necrosis is a major cause of death. Therefore, bacterial overgrowth and subsequent bacterial translocation can be prevented by administration of selected probiotics, because the usage of these bacterial supplementation have been shown to reduce infectious complications in patients with severe acute pancreatitis (187). The oral administration of *Lactobacillus plantarum* in combination with enteral feeding improved gut permeability and led to a significantly better clinical outcome (188). Treatment of severe acute pancreatitis could be adjusted by enteral nutrition (EN) and ecoimmunonutrition (EIN), because alone as well as in combination, both decrease the expression of plasma endotoxin, TNF, IL-6, bacterial translocation and pancreatic sepsis (189). As described in this review, synbiotics could prevent dysbiosis of the human gut, but administration of synbiotics may not affect the intestinal permeability in critically ill patients (150). A further clinical study with 72 patients have found the influence of pro- and synbiotics (termed as Synbiotic 2000FORTE) (*P. pentoseceus* 5-33:3, *L. mesenteroides* 32-77:1, *L. paracasei ssp*. 19, *L. planatarum* 2362) on the immune response in patients with multiple injuries (190). A significant decrease (p = 0.028) have been shown in the incidence of septic events as well as the occurrence of ventilation associated pneumonia by *Acinetobacter baumannii*. The risk of sepsis as a consequence of bacteremia was significantly decreased and even the treatment with the specific synbiotics prolonged the time of progression of primary bacteremia, compared to the placebo group (190). In the molecular level, white blood cell counts and serum C-reactive protein were significantly lower in patients treated with Synbiotic 2000FORTE compared to the placebo cohort and it could reduce the incidence of death caused by MODS (190).

## Conclusion

The impact of sepsis on the gut is manifold, e.g., sepsis mediated alteration of the gut-blood barrier and increase in the intestinal permeability, which may correlate with the phenomena of bacterial translocation and lymphatic activation (“toxic-lymph”). Systemic consequences of sepsis are widespread and concern to the coagulative system, the microbiome as well as enzymes, such as pancreatic proteases, MMPs and IAPs. Nevertheless, the therapeutic approaches for modulating the mucosal immune system are still rarely effective in daily routine. Recent published studies showing that treatment with FMT, probiotics and synbiotics are new concepts for gut-specific therapeutic prevention of sepsis (Table 1). Since the past decade, several clinical trials have been completed and are underway to comprehensively actualize the currently understood putative effectiveness of targeting the gut during sepsis. This has been presented in Table 1, enlisting all completed published, completed unpublished and ongoing trials so far. One exemplary study was proven to be an effective synbiotic treatment of fructooligosaccharides and *Lactoacillus plantarum* to preterm neonates which prevented sepsis and mortality in the treatment group (181). However, these promising therapeutic approaches are yet to be appraised as accepted therapeutic options. More clinical investigations could help substantiate these findings and extend them into becoming alternative treatment options. This also brings into light the importance of understanding the gut mucosal immune system, where further investigation is required to evaluate unknown sepsis-induced intestinal pathophysiological processes. Scarce as of now, nonetheless, investigations to understand the MIS would prove additionally beneficial so as to identify added novel therapeutic modalities.

**Table 1:** Table with ongoing and/or completed clinical trials targeting the gut in sepsis patients.

**Trial registration number and Name**	**Study Population and inclusion criteria**	**Short description of intervention and duration of treatment**	**Study duration, study endpoints**
NCT02127749 The role of the gut microbiota in the systemic immune response during human endotoxemia (MISSION-2) (168)	16 persons • Healthy • Caucasian • Non-smoking • Male • Age 18–25	Treatment of healthy male candidates with broad-spectrum antibiotics (ciprofloxacin, vancomycin, metronidazole) or placebo for 7 days. After a 36 h wash-out period, an i.v. bolus infusion of LPS given.	• Day 0 = 1 day before antibiotic treatment • Day 9 = 1 day after finishing treatment with broad-spectrum antibiotics, before i.v. LPS administration • Day 10 LPS administration (*t* = 0) • Blood sampled at *t* = 0,5; 1; 1,5; 2; 3; 4; 6; 8 h after LPS injection.
“*Bacillus clausii* for Prevention of late-onset sepsis in preterm infants: a randomized controlled trial” (184)	• Preterm neonates <34 weeks • 120 neonates grouped under “extreme preterm” • 124 grouped as “very preterm.”	Neonates of each group were randomized to receive prophylactic probiotic supplementation with *Bacillus clausii* or placebo till 6 weeks of postnatal age.	• Study started on 01.03.2012 and ended on 28.02.2014 • Study endpoint: End of 6 weeks supplementation of probiotics, or till discharge, or death, or occurrence of late-onset sepsis.
CTRI/2009/000945 Effect of oral glutamine supplementation on gut permeability and endotoxemia in patients with severe acute pancreatitis: a randomized controlled trial (191).	80 persons (age 18–80 years) *n* = 41 treatment *n* = 39 for placebo • Patients with acute severe pancreatitis • 3-fold increase in the levels of serum amylase. • Confirmed 1 or more organ failure and/or APACHE II > 8 and/or CT severity index> 7	Oral glutamine administration for 7 days. Objective of the study was to check for the effect of glutamine supplementation on endotoxemia and gut permeability following severe acute pancreatitis.	• Started on November 2009 and ended on October 2012. • Duration of intervention has been 7 days • Day 0 = start with the intervention • Endpoint day 7 = end of intervention with glutamine administration
ISRCTN61157513 Modulation of gut function using Gut Specific Nutrients in the critically ill (192).	50 persons *n* = 25 treatment *n* = 25 for placebo Critically ill surgical patients with inadequate gut function.	Treatment with a gut-specific nutrients cocktail or Placebo-cocktail for 30 days	• Restoration of gut function to normalcy was the primary endpoint. • Patients reviewed twice daily for 1 month of their hospital stay then followed-up till 3 months.
NCT00518596 Prevention of infection in Indian neonates—phase II probiotics study (181).	4,556 infants as *n* = 2,278 synbiotic; *n* = 2,278 placebo • Neonate >24 h and <96 h old • Birth weight >2,000 g • Breastfeeding begun by 24 h of life • Ability to tolerate oral feeds.	Daily one-time oral synbiotic treatment with 10 billion *Lactobacillus plantarum* and 150mg of fructoologiosaccharide for 7 days from day 2-4 of post-natal life.	Study started on October 2008 and ended on May 2012 Duration of clinical surveillance was 60 days of post-natal life with daily evaluation of the infants
R000007633 Synbiotics modulate gut microbiota and reduce enteritis and ventilator-associated pneumonia in patients with sepsis: a randomized controlled trial (179).	72 Patients; *n* = 35 synbiotics; *n* = 37 placebo • Age >16 years • > 3 days of mechanically ventilated sepsis patients	Daily oral administration of synbiotics (*Bifidobacterium breve* strain Yakult, *Lactobacillus casei* strain) Shirota and (galactooligosaccharide) 3 days post hospital admission.	4 weeks after admission to hospital, study was ended. • **Primary outcome** within 4 weeks: ventilator induced pneumonia, bacteremia and enteritis. • **Secondary outcomes**: fecal bacterial numbers, concentration of organic acid and mortality within 4 weeks.
“Synbiotics decrease the incidence of septic complications in patients with severe sirs: a preliminary report (193).”	55 patients *n* = 29 synbiotics *n* = 26 non-synbiotics • Severe SIRS • C-reactive protein >10 mg/dl.	Oral treatment with synbiotics, *Bifidobacterium breve* (3 × 10^8^/day), *Lactobacillus casei* and galactooligosaccharide (10 g/day) or placebo on day of admission.	• Study started on July 2004 and ended on March 2005 • Study endpoint has been defined as the last collection of fecal samples on day 40 after admission.
NCT00835874 Probiotic administration to mothers of preterm infants to prevent necrotizing enterocolitis and sepsis. (194)	57 participants • Mothers of preterm infants Pumping breast milk	Oral treatment with probiotics *Lactobacillus acidophilus, Bifidobacterium lactis* once daily	**Primary Outcome Measures**: 3 month evaluation of all-cause mortality, incidence and severity of necrotizing enterocolitis and appearance of bacterial or fungal infection (blood, csf, or urine cultures).
NCT01100996 Anti-inflammatory effects of enriched enteral nutrition during human experimental endotoxemia (VIHE) (195).	36 participants • Age >18 and <35 • Male Non-smoking	Triggering of human endotoxemia by LPS infusion. Then:• No intervention—fasted control or, • Placebo comparator—control feeding (20% fat, 16% protein and 49% carbohydrates) 1 h after LPS administration until 6 h after LPS or, • Active comparator— enriched feeding (46% fat, 24% protein and 30% carbohydrates and phospholipids) 1 h and until 6 h after LPS administration.	• **Primary Outcome Measures:** Circulating cytokines (Time Frame: several time points from LPS administration until 24 h) • **Secondary Outcome Measures:** Markers for sub-clinical organ damage (kidney, endothelium, intestine) (Time Frame: several time points from LPS administration until 24 h)
NCT02768324 The effect of gut microbiota on the prognosis of sepsis. (180)	30 participants • Age >18 and <65 Sepsis	No treatment, only observation of septic patients and collecting stool and blood samples.	Primary Outcome: Discharged from ICU; Time Frame 7 days
NCT03472170 Clinical trial for valuation of the effectiveness of lactoferrine in the prevention of sepsis in new premature born. bimonitorization of the antinflammatory mechanisms, antioxidants and the intestinal microbiote. (LACTOPREM)	26 participants • Male or female children born preterm • Birth weight <1,500 g and or EG <32 weeks	Enteral administration of bovine lactoferrin or treatment with placebo for 4 weeks (exceptional extending for 6 weeks in newborns EG <28 weeks or birth weight <1,000 g)	Primary Outcome: Incidence of proven and probable late sepsis (12 months)
NCT03488940 Effect of different feeding method on gastrointestinal function of septic patients (DFM-GF Trial) (DFM-GF)	60 participants • Sepsis • APACHE-II Score >15 points	Patients were allocated into 3 cohorts• 24 h group • 16 h group • intermittent group Each group refers to the time upto which nutrition preparation is enterally pumped. The duration of the treatment is 7 days.	• **Primary Outcome Measures:** The mean time (hours) that reach to the caloric goal in every group (7 days after intervention) • **Secondary Outcome Measures:** The rate of onset Gastric residual (%) (baseline and first 7 days after intervention); Abdominal pressure mmHg; Level of plasma motilin (pg/ml); Rate of new onset pneumonia.
NCT03861325 acute intestinal failure in critically ill patients and microbial translocation (IGA-TM)	50 participants • Age >18 • Septic shock (as defined by the 3rd International Conference for the definition of sepsis and septic shock)	Observation for 7 days—no treatment	• **Primary Outcome Measures:** Incidence of microbial translocation in critical illness by measuring 16S rDNA and 18S rDNA plasma levels (at admission, 12 h, 1 d, 2 d, 3, and 7 d after admission to hospital) • **Secondary Outcome Measures:** Plasma I-FABP and zonulin (at admission, 12 h, 1 d, 2 d, 3, and 7 d after admission to hospital); Correlation between titer of I-FABP, zonulin, 16S rDNA, 18S.
NCT02306239 The effect of terlipressin on intesitnal function in septic shock patients	40 participants • Age >18 and <80 • Septic shock	Treatment with Terlipressin or Norepinephrine	**Primary Outcome Measure:** Evaluation of the intestinal function based on abdominal distension, intestinal bleeding, peritonitis, plasma DAO level, and enteral nutrition (Time Frame 7 days).
NCT00378586 Barrier function and production of inflammatory cytokines in the rectal mucosa in patients with septic shock	30 participants • Age >18 • Septic shock	Extraction of a rectal biopsy	**Primary Outcome Measure:** • Measurement of IL-6 and TNF levels in the rectal mucosa • Measurement of rectal lactic acid

*Various clinical trials have enabled active application of prebiotic, probiotic and synbiotic treatment modalities incorporated into traditional therapeutic approaches. Here is a comprehensive tabular representation of all clinical trials that have employed such alternative treatments in the context of sepsis related modulation of the gut. Many of these clinical trials have been instrumental in understanding the ensuing gut pathophysiological changes, its effect on SIRS and how the altered microbiome is a robustly affected cause and effect thereof. i.v., intravenous; LPS, lipopolysaccharide; LGG, Lactobacillus GG; ICU, intensive care unit; SIRS, systemic inflammatory response syndrome; CRP, C-reactive protein; SOFA, sequential organ-failure assessment; IL-6, interleukin 6; APACHE-II score, Acute Physiology And Chronic Health Evaluation- II Score; mmHG, millimeter of mercury; DAO, Diamine Oxidase; I-FABP, intestinal fatty acid-binding protein; rDNA, ribosomal deoxyribonucleic acid; TNF, tumor necrosis factor; csf, cerebrospinal fluid; EF, ejection fraction*.

## Author Contributions

FH and MH-L conceptualized the review. FH and SC performed the literature search for the review. FH wrote the review as a first author, with written contribution by SC as the second, RH as the third and MH-L as the senior author. The table was compiled and prepared by FH and SC. Figures were prepared with the help of Dr. Stephanie Denk as mentioned in Acknowledgments.

### Conflict of Interest Statement

The authors declare that the research was conducted in the absence of any commercial or financial relationships that could be construed as a potential conflict of interest.

## Abbreviations

AMP: antimicrobial peptide
APC: antigen presenting cell
APACHE-II: Acute Physiology and Chronic Health Evaluation- II Score
Bcl-2: B-cell lymphoma gene-2
C-BF: cathelicidin-BF
C3: complement factor 3
C3a: activated complement factor 3
C5: complement factor 5
CD4: cluster of differentiation 4
CLP: cecal ligation and puncture
COX-2: cyclooxygenase-2
CRP: C-reactive protein
CSF: cerebrospinal fluid
DAMP: danger-associated molecular pattern
DAO: Diamine Oxidase
DC: dendritic cell
DIC: disseminated intravascular coagulopathy
DNA: deoxyribonucleic acid
EF: ejection fraction
FXIII: clotting factor XIII
γ-EV: dietary dipeptide gamma-l-glutamyl-l-valine
GALT: gut-associated lymphoid tissue
HMGB1: high-mobility-group-protein-B1
IAP: intestinal alkaline phosphatase
IBD: inflammatory bowel disease
ICAM: intercellular adhesion molecule
ICU: intensive care unit
IEC: intestinal epithelial cell
IEL: intraepithelial lymphocyte
I-FABP: intestinal fatty acid-binding protein
IFN: interferon
IgA: immunoglobulin A
IL: interleukin
IκBα, nuclear factor of kappa light polypeptide gene enhancer in B-cells inhibitor: alpha
i. v.: intravenous
LPS: lipopolysaccharide
LGG: Lactobacillus GG
M-cells: microfold cells
MALT: mucosa-associated lymphoid tissue
MIS: mucosal immune system
MLCK: myosin-light chain kinase
mmHG: millimeter of mercury
MMP: matrix metalloprotease
MODS: multiple organ dysfunction syndrome
MOF: multi-organ failure
MUC: mucin
NF-κB: nuclear factor kappa-light-chain-enhancer of activated B-cells
NOD: nucleotide-binding oligomerization domain
PAI-1: plaminogen activator inhibitor-1
PAMP: pathogen-associated molecular pattern
PG: proteoglycan
PRR: pattern recognition receptor
rDNA: ribosomal deoxyribonucleic acid
RNA: ribonucleic acid
ROS: reactive oxygen species
SATI: short acting thrombin (factor II) and factor Xa (FXa) inhibitor
SIRS: systemic inflammatory response syndrome
SIRT2: NAD-dependent deacetylase sirtuin 2
SOFA: sequential organ-failure assessment
SP: surfactant protein
sTNF: soluble TNF
TAB: TAK1-binding protein
TCR: T-cell receptor
TED: transepithelial dendrites
TF: tissue factor
Th: T helper lymphocyte
TJ: tight junction
TLR: Toll-like-receptor
TNF: tumor necrosis factor
TRAF: TNF receptor associated factor
VCAM: vascular cell adhesion protein
ZO-1: zonulin-1.
